# The relationship between regional abdominal fat distribution and both insulin resistance and subclinical chronic inflammation in non-diabetic adults

**DOI:** 10.1186/1758-5996-6-49

**Published:** 2014-04-01

**Authors:** Ching-Jung Hsieh, Pei-Wen Wang, Tse-Ying Chen

**Affiliations:** 1 Department of Internal Medicine, Division of Endocrinology and Metabolism, Kaohsiung Chang Gung Memorial Hospital, Chang Gung University College of Medicine, 123 Ta-Pei Road, Niao- Sung Hsiang, Kaohsiung Hsien 83305, Taiwan

**Keywords:** Subcutaneous fat, Intra-peritoneal fat, Retroperitoneal fat, Insulin resistance, High sensitive C-reactive protein, Adiponectin

## Abstract

**Objective:**

Obesity is associated with a high risk of insulin resistance (IR) and its metabolic complications. It is still debated that distributions of adipose tissue relate to an excess risk of IR and chronic inflammation in different race. This study was designed to examine the relation between insulin sensitivity, chronic inflammation and central fat distribution in non-diabetic volunteers in Taiwanese.

**Methods:**

There were 328 volunteers without family history of diabetes mellitus and with normal oral glucose tolerance test enrolled. Total body fat and abdominal fat were measured. Abdominal fat was categorized into intraperitoneal (IP), retroperitoneal (RP) and subcutaneous (SC) fat. The IR index was estimated by homeostatic model assessment. Five inflammatory markers: adiponectin, leptin, tumor necrosing factor-α (TNF-α), resistin and high sensitive CRP (hs-CRP) were measured.

**Results:**

IR was related to IP fat (r = 0.23, *p* < 0.001), but not RP fat, SC fat or total body fat. After correcting for age and sex, IP fat was the only significant predictor of IR (r2 = 58%, *p* = 0.001). Leptin showed the strongest relationship with all fat compartments (IP fat: r = 0.44, *p* = 0.001; RP fat: r = 0.36, *p* = 0.005, SC fat: r = 0.54, *p* < 0.001; total body fat: r = 0.61, *p* < 0.001). The hs-CRP and adiponectin were closely related both to IP (r = 0.29, *p* = 0.004; r = -0.20, *p* = 0.046, respectively) and total body fat (r = 0.29, *p* = 0.004; r = -0.29, *p* = 0.005, respectively), but not RP, or SC fat. TNF-α and resistin were not correlated to any fat compartment. After correcting for age and sex, leptin variance was mostly explained by SC fat (41.3%), followed by IP fat (33.6%) and RP fat (25.3%). The hs-CRP and adiponectin variance were mostly explained by IP fat (40% and 49% respectively).

**Conclusions:**

IP fat is better predictors of IR and subclinical chronic inflammation in Taiwanese adults. A disproportionate accumulation of abdominal fat is associated with increased risk of cardiovascular diseases.

## Introduction

The prevalence of obesity and overweight are still increasing globally. Compared to other countries of Asian, Taiwan has a higher percentage of people who are obese than Australia, Germany, New Zealand and the UK. In 2005, the prevalence of obesity and overweight in Taiwan are 19.2% and 30.5% in men, 13.4% and 21.3% in women [1]. Central obesity, as waist circumference, is clearly related to high risk of insulin resistance and the associated metabolic complications. From most studies, a more abdominal distribution of fat is associated with type 2 diabetes mellitus and worse outcome of cardiovascular disease [2]. The abdominal fat includes subcutaneous (SC) fat of abdomen, intraperidonial (IP) fat and retroperidonial (RP) fat. However, most studies revealed the metabolic complication with visceral fat and SC fat of abdomen [3,4]. There are few studies clearly investigate the relationship of metabolic complication with these three reginal fat distribution and separate viseral fat to IP fat and RP fat, eapecially in Asian. How adipose tissue causes metabolic abnormality is often linked with adipokines and other inflammation markers [5,6]. The most well studied adipokines are leptin and adiponectin. Resistin and TNF-α secreted by adipose tissue may also play roles on regulate insulin resistance [7]. However, how the regional distribution of abdominal adipose tissue affects this adipokines is still controversial in different race. Most studies verified visceral area fat (also known as intra-abdominal fat) is strongly associated with insulin resistance but seldom analyze the fat distribution in the abdomen. Presence of brown adipose tissue, a metabolically highly active organ with increased thermogenic activity, in RP fat is always neglected. As for subcutaneous fat, the correlation to insulin resistance is still debated [8-11]. These inconsistent results are likely because of marked differences in ethnicity of the cohorts studied.

On account of different ethnicity may have different distribution of abdominal fat, in this study, we investigate regional fat deposition of abdomen, homeostatic model assessment for insulin resistance (HOMA-IR) and chronic inflammation factors in healthy non-diabetic Taiwanese adults with large population. The relationship between regional fat deposition and both insulin resistance and chronic inflammation are analyzed. This study was designed to examine the relation between insulin sensitivity, chronic inflammation and central fat distribution in non-diabetic volunteers in Taiwanese.

## Materials and methods

### Subjects

There were 328 volunteers enrolled to participate in the study. They all had normal blood glucose (179 women and 149 men aged 41 to 59 years; mean ± SD, 49.2 ± 9.0 years) defined by fasting blood glucose less than 100 mg/dL and normal oral glucose tolerance test. Subjects included in this study were all over 40 years old, ethnically Chinese, and from the same region in Taiwan at the time of study. The exclusion criteria were diabetes, hypertension, chronic alcohol drinking and family history with diabetes mellitus, determined by taking a history. Four measurements were taken of all subjects after fasting for more than 8 hours (fasting blood glucose and insulin levels, total body fat, and abdominal fat measurement). Five inflammatory markers: adiponectin, leptin, tumor necrosing factor-α (TNF-α), resistin and high sensitive CRP (hs-CRP) were also measured. The Medical Ethics and Human Clinical Trial Committee of Chang Gung Memorial Hospital approved the study, and all subjects provided written informed consent before participating.

### Methods

**Figure 1:**
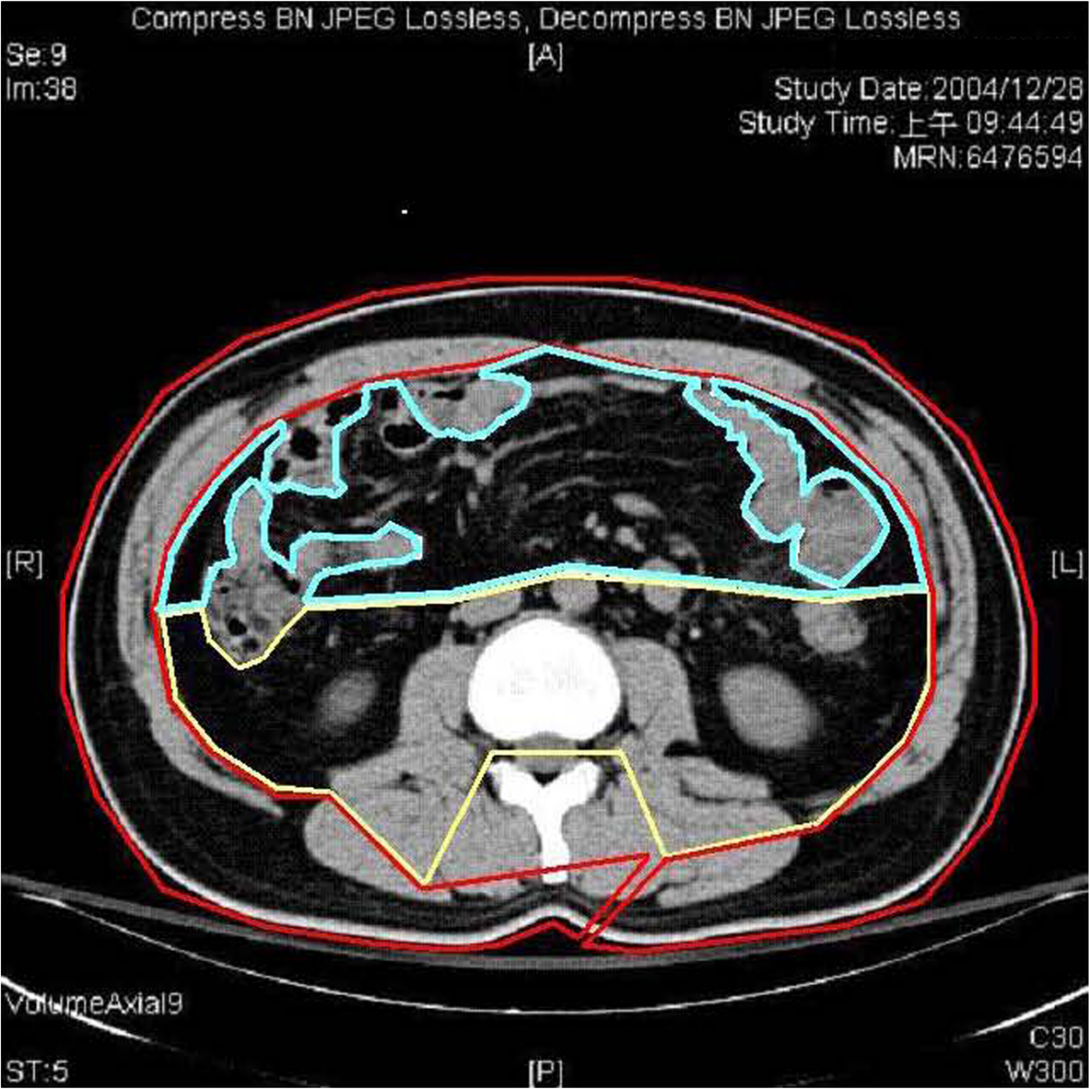
The CT of abdomen shows measurement of intra-peritoneal fat (blue line), retro-peritoneal fat (yellow line) and subcutaneous fat (red line).

Total body fat mass was measured by electrical bioimpedance analysis (BIA), tetra-polar vertical BIA using 8-point tactile electrode (InBody 230, BioSpace Inc., Los Angeles, CA, USA). All subjects were tested after eight-hour fast while wearing light clothing and empting urinary bladder. Abdominal fat was measured by computed tomography (CT). The average Hounsfield values from five 3-mm-slices was calculated. Abdominal fat was categorized into three distinct compartments, the intra-peritoneal, retroperitoneal and subcutaneous fat compartments. These were measured at an attenuation range of -150 to -50 Hounsfield units at the L1-2 vertebral disk level. Distinction between retroperitoneal and intra-peritoneal fat areas was defined by drawing straight lines from the mid-point between the abdominal aorta and the inferior vena cava through the centers of the descending and ascending colon (Figure 1).

### Assay

The plasma concentrations of glucose (Hitachi 7250 Special auto-analyzer; Hitachi, Tokyo, Japan) and insulin (Access automated immunoassay; Beckman Instruments, Fullerton, CA) were measured. Insulin resistance (IR) was determined by homeostasis model assessment (HOMA) and calculated using fasting plasma glucose and fasting insulin levels in each participant, as follows: HOMA IR = fasting glucose (mmol/l) × fasting insulin (mU/ml)/22.5.

Inflammatory markers: adiponectin, TNF-α, and resistin were checked with commercially available ELISA kits (R&D Systems Inc). The lower limit of fasting adiponectin detection with this assay was 0.89 ng/ml. The intra-assay coefficients of variation were 2.5, 3.4, and 4.7% at low (19.8 ng/ml), median (69.9 ng/ml) and high (143 ng/ml) levels, respectively. The inter-assay coefficients of variation were 6.8, 5.8 and 6.9% at low (20.5 ng/ml), median (74.4 ng/ml) and high (157 ng/ml) levels. The lower limit of resistin concentration detection with this assay was 0.055 ng/ml. The intra-assay coefficients of variation were 5.0, 5.3, and 3.8% at low (0.60 ng/ml), median (2.26 ng/ml) and high (4.72 ng/ml) levels. Inter-assay coefficients of variation were 8.2, 9.2 and 7.8% at low (0.61 ng/ml), median (2.28 ng/ml) and high (4.72 ng/ml) levels. The lower limit of TNF-αconcentration detection with this assay was 0.5-5.5 pg/ml. The intra-assay coefficients of variation were 5.2, 4.2, and 4.6% at low (48.1 pg/ml), median (317 pg/ml) and high (587 pg/ml) levels. The inter-assay coefficients of variation were 7.4, 4.6 and 5.4% at low (45.8 pg/ml), median (301 pg/ml) and high (587 pg/ml) levels, respectively.

The serum hs-CRP level was measured using a high-sensitivity% monoclonal antibody assay (Diagnostic Systems Laboratories, Inc). The run-to-run CVs, at hs-CRP concentrations of 19.6, 182.9, and 300.4 ng/ml were 3.6, 3.9, and 2.1%.

Leptin was determined by radioimmunoassay (Human Leptin RIA kit, Linco Research, MO, USA). Intra-assay coefficients of variation were 8.3, 3.9, and 3.4% at low (4.9 ng/ml), median (10.4 ng/ml) and high (25.6 ng/ml) levels. The inter-assay coefficients of variation were 6.2, 4.7 and 3.6% at low (4.9 ng/ml), median (10.4 ng/ml) and high (25.6 ng/ml) levels. Analytical sensitivity remained at a level of around 0.5 ng/ml.

All statistical analyses were performed using the Statistical Package for Social Science program (SPSS for Windows, version 11.5; SPSS, Chicago, IL). Continuous variables were expressed as means ± SD. The relationships in variable pairs were analyzed by Person correlation coefficients and stepwise multiple regression analyses. The level of significance was taken as *P* = less than 0.05.

## Results

Study group characteristics are given in Table 1.

**Table 1:** The characteristics of the study group

	**Minimum**	**Maximum**	**Mean**	**Standard deviation**
**Age (year-old)**	**41**	**59**	**49.20**	**9.00**
BMI	17.51	39.26	28.58	10.27
Body fat (%)	13.00	41.20	27.66	14.75
Intra-peritoneal fat (cm^3^)	7550.00	56835.40	29188.67	21382.91
Retro-peritoneal fat (cm^3^)	2243.32	46774.30	24433.28	22231.98
Subcutaneous fat (cm^3^)	59741.12	134556.08	95929.75	36293.45
IR (HOMA)	9.54	16.87	13.57	2.24
hs-CRP (ng/ml)	0.25	13.09	6.84	6.05
Adiponectin ((μg/ml))	22.28	117.53	65.28	38.68
Leptin (ng/ml)	1.62	33.79	15.71	13.66
TNF-α (pg/ml)	6.50	19.42	13.66	5.75
Resistin (ng/ml)	2.16	49.82	25.28	20.36

*BMI* body mass index, *IR* insulin resistance, *HOMA* homeostasis model assessment, *TNF-α* tumour necrotic factor -α.

### Relation between fat compartments and insulin resistance

**Figure 2:**
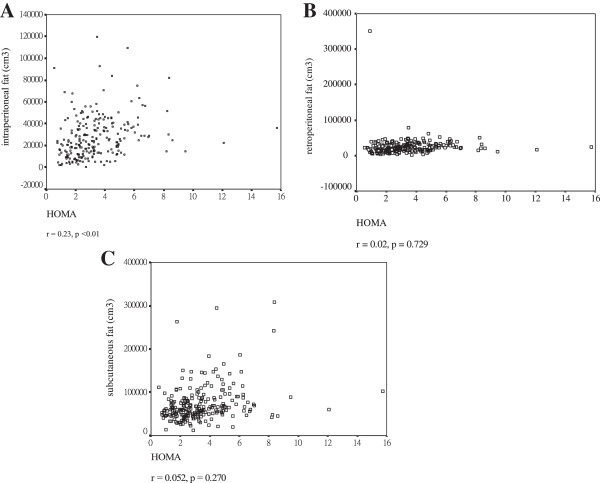
**The relation between insulin resistance index calculated by HOMA and body fat distribution. A**. Highly correlation between intra-peritoneal fat and insulin resistance. **B**. No significant correlation between retro-peritoneal fat and insulin resistance. **C**. No significant correlation between subcutaneous fat and insulin resistance.

The IP fat compartment was significantly related to HOMA IR (r = 0.23, *p* < 0.00, Figure 2A). The RP fat, SC fat compartment and total body fat showed no relationship to HOMA IR (r = 0.022, *p* = 0.729; r = 0.052, *p* = 0.27, r = 0.18, *p* = 0.075, respectively, Figure 2B and 2C). Stepwise regression analysis showed that the IP fat compartment was the only significant predictor of insulin resistance (r2 = 58%, *p* = 0.001).

### Relations between fat compartment and chronic inflammation markers after correct for age and sex

In the Table 2, the hs-CRP was closely related to IP fat (r = 0.29, *p* = 0.004) and total body fat (r = 0.29, *p* = 0.004), but not to the RP fat or SC fat (r = 0.20, *p* = 0.084; r = 0.13, *p* = 0.216, respectively).

**Table 2:** Correlation between fat distribution and chronic inflammatory markers (correcting for age and sex)

**Fat distribution**	**Intra-peritoneal fat (cm**^**3**^**)**	**Retro-peritoneal fat (cm**^**3**^**)**	**Subcutaneous fat (cm**^**3**^**)**	**Total body fat (cm**^**3**^**)**
**Markers**				
Hs-CRP (ng/ml)	r = 0.29, p = 0.004	*r = 0.20, p = 0.084*	r = 0.13, p = 0.216	r = 0.29, p = 0.004
Adiponectin (μg/ml)	r = -0.20, p = 0.046	r = -0.13, p = 0.222	r = 0.19, p = 0.335	r = -0.29, p = 0.005
leptin (ng/ml)	r = 0.44, p < 0.001	r = 0.36, p = 0.005	r = 0.54, p < 0.001	r = 0.61, p < 0.001
TNF-α (pg/ml)	r = 0.05, p = 0.666	r = -0.36, p = 0.171	r = 0.08, p = 0.886	r = -0.08, p = 0.403
resistin (ng/ml)	r = 0.08, p = 0.427	r = 0.03, p = 0.740	r = 0.05, p = 0.654	r = 0.03, p = 0.806
HOMA	r = 0.23, p < 0.001	r = 0.02, p = 0.729	r = 0.05, p = 0.270	r = 0.18, p = 0.075

Leptin showed the strongest relationship with all fat compartments (IP fat: r = 0.44, *p* = 0.001; RP fat: r = 0.36, *p* = 0.005, SC fat: r = 0.54, *p* < 0.001; total body fat: r = 0.61, *p* < 0.001, Table 2). In a stepwise regression analysis, SC fat explained 41.3% of the variance of leptin levels, IP fat explained 33.6%, and the RP fat explained 25.3%.

Adiponectin was closely related to IP fat (r = -0.20, *p* = 0.046) and total body fat (r = -0.29, *p* = 0.005), but not to the SC and RP fat (r = 0.19, *p* = 0.335; r = -0.13, *p* = 0.222, respectively, Table 2). In a stepwise regression analysis, the IP fat explained 49% of the variance of adiponectin levels.

TNF-α and resistin were not correlated to all fat compartment.

## Discussion

In this study, we found the relationship between regional abdominal adiposity and insulin resistance in a group of non-diabetic, middle-aged Taiwanese with varying degrees of body mass index. IP fat mass is the best predictor of insulin resistance. In our study, we used CT for adipose tissue evaluation and also for multiple compartments of body measurement [12]. A strong relationship was noted between IP fat and insulin resistance.

In clinical practice, waist circumference as central obesity was used and correlated waist circumference with metabolic syndrome. However, waist circumference is composed of abdominal SC fat, and intra-abdominal fat. Intra-abdominal adipose tissue (visceral fat) is further separated to IP fat and RP fat, a distinction which has been validated in human cadavers [13]. Previous work had verified that most of the intra-abdominal adipose tissue mass is IP (61–71%) and a much smaller element is RP (29–33%) [13]. **Marin** et al’s study has shown IP fat, rather than RP or SC fat, more strongly correlated with systemic metabolic variables (e.g., plasma insulin, blood glucose levels, glucose disposal rate, and systolic blood pressure). There is a higher turnover of lipids in visceral than in the other fat depots, which is closely correlated to systemic insulin resistance and glucose metabolism [14]. However in the study of Abate et al., subcutaneous truncal fat plays a major role in obesity-related insulin resistance in men, whereas IP fat and RP fat have a lesser role [15]. In this study only 39 healthy men were included. The discrepancy may be due to different body fat distribution in different gender, age and ethnicity. For example, multiple insulin resistance risk factors occur in apparently healthy Asian Indians with normal body mass index (BMI) [16-18]. Altered body composition with greater abdominal fat is associated with insulin resistance, hyperinsulinemia, and dyslipidemia in this population. When BMI and body fat (BF%) were studied in Taiwan, Taiwanese subjects had a relatively lower BMI but a higher BF% than Caucasians. In general, the newly proposed Asia-Pacific BMI cutoffs for overweight (> or =23 kg/m2) and obesity (> or =25 kg/m2) may be acceptable to both male and female Taiwanese subjects. The corresponding BF% cutoffs for obesity would be 25% in male and 38% in female Taiwanese subjects, respectively [19]. In our study, we found that IP fat compartment was the only fat compartment that significantly correlated to HOMA IR. SC fat, which is controversial in previous studies, is not an independent factor of insulin resistance in our studies. This allows us to specifically determine which compartment of fat is of primary importance in the etiology of insulin resistance in Taiwanese people.

Many recent studies revealed that chronic inflammation participates in the development of metabolic syndrome related coronary artery disease. These prospective studies also showed an association between central obesity (by waist circumference) and chronic inflammation [20-22]. The hs-CRP value can also be used as an independent predictor of coronary heart disease. The American Heart Association identified this marker as the best clinical detector for cardiovascular disease [23]. Published data also show that visceral adipose tissue is strongly associated with circulating levels of CRP [5,24,25]. In our study, the hs-CRP is highly related to IP fat and total body adipose tissue but not RP and SC fat. Here, we further specified that IP fat appears to be the strongest predictor for high hs-CRP.

Leptin, TNF-α, resistin and adiponectin are adipose tissue secretary proteins. Leptin, TNF-α, and resistin correlating with body fat mass, has been reported to be associated with insulin resistance and atherosclerosis [26,27]. On the other hand, adiponectin has antidiabetic and antiatherogenic properties [28]. Our previous work has confirmed that during weight reduction programs adiponectin plasma levels increase while leptin levels decrease [29]. Leptin levels were thought correlated with total and subcutaneous adipose tissue, but not with visceral adipose tissue [16,30]. However, in one study investigating South Asian in American, leptin is strongly correlated with both SC fat and visceral fat [31]. In this Taiwanese group, our data clearly show that plasma leptin is associated with all part of adipose tissue, but still the primary relationship is with subcutaneous fat. Therefore, for Taiwanese or Asian, leptin may not be the best predictor for metabolic syndrome, only for general obesity alone. As for adiponectin, our study showed adiponectin level was inversely related to IP fat, but not associated with the SC and RP fat. This result is similar to two studies in different ethnicity [32,33]. We separated intra-abdominal fat (visceral fat) to intra-pertonium and retro-peritonium, which revealed adiponectin more related to IP fat than fat mass of other area and may be the negative predictor for metabolic syndrome.

TNF-α is an adipokine involved in systemic inflammation and is a member of a group of cytokines that stimulate the acute phase reaction. However, obesity, especially IP fat accumulation needs long-term changes. Although previous study revealed that TNF-α increases leptin gene expression and circulating leptin levels [34], some data revealed no correlation found between TNF-α with any fat distribution [5,35]. In Beasley et al’ study, this large across racial study demonstrates visceral fat is only positively associated with TNF-α except in black men but not white men [36]. These different results may indicate ethnicity is important in characteristic of fat compartment again. Resistin, a cysteine-rich 12.5 kDa adipocytokine with a controversial history regarding its role in the pathogenesis of obesity-mediated insulin resistance and type 2 diabetes mellitus in rodent models, is still controversial in the role of glucose metabolism in human, is probably involved in the regulation of inflammatory processes rather than in insulin sensitivity [34,37]. No relationship between fat distribution and resistin is also found in our study. In our results, the amount of IP fat was significantly positive correlated with hs-CRP and leptin but negative correlated with adiponectin. IP fat may be inherently different from RP and SC fat, e.g., in cellular composition, tissue dynamics, adipokine release, and hormonal responses. The “portal theory” also assumes inflammatory factors released from IP fat into the portal vein directly contributes to hepatic insulin resistance [38].

In conclusion, the findings of this study confirm that IP fat is a strong predictor of insulin resistance and metabolic syndrome in Taiwanese. These observations reinforce the importance of treatment strategies designed to reduce IP part rather than RP part of visceral fat. Our results also suggest a relationship between IP adiposity and the chronic inflammation process. This holds irrespective of age or other potential confounders, and was more prominent than the relationship between total obesity. It may be that, through the inflammation process and adipokines, a disproportionate accumulation of IP fat is associated with increased coronary risk.

## Competing interests

There are no competing interests relevant to this article to report.

## Authors’ contributions

P-WW, T-YC and C-JH collected data from their patients. C-JH performed the data analysis and wrote the manuscript. All authors read and approved the final manuscript.
